# Retrospective study of intravascular large B-cell lymphoma cases diagnosed in Quebec: A retrospective study of 29 case reports: Erratum

**DOI:** 10.1097/MD.0000000000007018

**Published:** 2017-05-19

In the article, “Retrospective study of intravascular large B-cell lymphoma cases diagnosed in Quebec: A retrospective study of 29 case reports”,^[1]^ which appeared in Volume 96, Issue 5 of *Medicine*, two of the cells in Table 4, “Leukemia” and “4(14)” appeared on the wrong row and should have appeared one lower. Please see the corrected table below.

## References

[1] Brunet V Marouan S Routy J-P Retrospective study of intravascular large B-cell lymphoma cases diagnosed in Quebec: A retrospective study of 29 case reports. *Medicine*. 2017 96:e5985. 28151891 10.1097/MD.0000000000005985 PMC5293454

