# Retrospective study of intravascular large B-cell lymphoma cases diagnosed in Quebec: A retrospective study of 29 case reports: Erratum: Erratum

**DOI:** 10.1097/MD.0000000000008227

**Published:** 2017-09-29

In the erratum, “Retrospective study of intravascular large B-cell lymphoma cases diagnosed in Quebec: A retrospective study of 29 case reports: Erratum”,^[1]^ which appeared in Volume 96, Issue 20 of *Medicine*, the corrected table was not included. Two of the cells in Table 4, “Leukemia” and “4(14)”, appeared on the wrong row and should have appeared one lower. Please see the corrected table below.

**Table 4:**
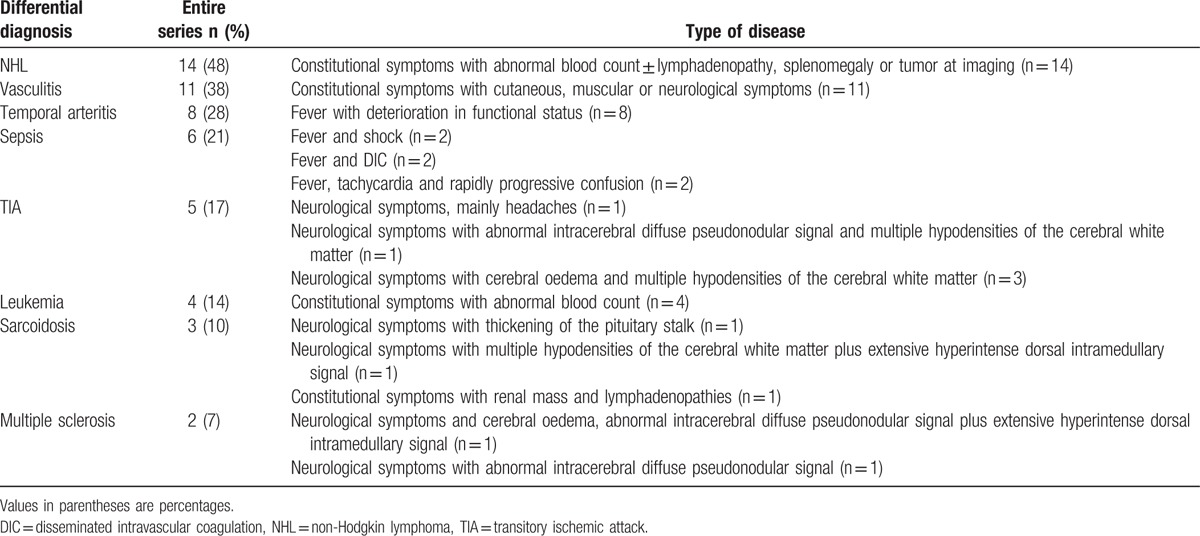
Differential diagnosis in the entire series (n = 29).
